# Dataset on adsorption of phenol onto activated carbons: Equilibrium, kinetics and mechanism of adsorption

**DOI:** 10.1016/j.dib.2020.106312

**Published:** 2020-09-14

**Authors:** Diego Felipe Hernández-Barreto, Liliana Giraldo, Juan Carlos Moreno-Piraján

**Affiliations:** a Departamento de Química, Facultad de Ciencias, Grupo de sólidos porosos y calorimetría, Universidad de los Andes, Bogotá, Colombia; b Departamento de Química, Facultad de Ciencias, Grupo de calorimetría, Universidad Nacional de Colombia, Bogotá, Colombia

**Keywords:** Adsorption, Activated carbon, Intraparticle diffusion, Film diffusion

## Abstract

Two activated carbons (AC) prepared from onion leaves (OL) *(Allium fistulosum)* and palm kernel shell (PS) *(Elaeis guineesis)* were used to adsorb phenol from aqueous solution. Adsorption kinetics was studied by *Pseudo-first order (PFO)* and *Pseudo-second order (PSO)* models, while equilibrium was modelled using Langmuir, Freundlich, Toth and Redlich Peterson isotherms. Adsorption mechanism was analyzed applying Boyd and intraparticle diffusion models. The parameters of each one of the models were calculated using Minitab17® by non-linear regression. Piecewise linear regression was applied to calculate the parameters of Boyd and intraparticle diffusion models. Phenol adsorption onto activated carbons is describe better by Langmuir isotherm and PSO kinetic model. Maximum adsorption capacity was between 30 and 40 mg.g^−1^.

## Specifications Table

**Table:** 

Subject	Physical Chemistry
Specific subject area	Surface chemistry and adsorption
Type of data	Table Figure
How data were acquired	Phenol concentration was determined using an UV–Vis Agilent Technologies Cary100 spectrophotometer. Isotherms and kinetic models were fitted using Microsoft Excel and Minitab17. Nitrogen physisorption isotherms at 77 K were obtained using Autosorb IQ2 Quantachrome Instruments.
Data format	Raw and Analyzed.
Parameters for data collection	N_2_ isotherms were obtained by measuring 31 adsorption points and 10 desorption points. Phenol adsorption isotherms were studied at 308 K, using an AC dosage of 2.5 g.L^−1^, neutral pH and varying concentrations between 5 and 200 ppm. Equilibrium was reached after two days in dark. Adsorption kinetics was studied at 308 K, 2.5 g.L^−1^ of AC, initial concentration of 100 ppm and neutral pH. Samples were taken at minutes 1, 4, 9, 16, 25 and 60 and every half an hour until seven hours passed.
Description of data collection	Phenol concentration was determined by UV–Vis at λ_max_ = 270 nm. Aliquots were taken and filtered with PTFE (0.22 μm) filters and then analyzed. Absorption data was transformed to phenol concentration using a calibration curve, and adsorption capacities were calculated by a mass balance.
Data source location	Departamento de Química, Facultad de Ciencias, Universidad de los Andes, Bogotá, Colombia.
Data accessibility	Data are provided with the article and in the Supplementary File.

## Value of the Data

• This data is useful because it provides information about phenol adsorption, specifically isotherms, kinetics and mechanism of adsorption, using two different activated carbons.
• Researches focused on surface chemistry and adsorption can be benefit from these data.
• This data can be used as a starting point for researchers that want to study adsorption equilibrium, kinetics and mechanism, or can be useful for comparison between different phenol adsorption systems.
• The additional value of this data is the use of non-linear regression and piecewise linear regression for fit different models, to study phenol adsorption over activated carbons.
• The models applied allow researchers to predict capacities, rates and limiting steps in phenol adsorption over activated carbons of different physicochemical properties.

## 1 Data Description

**Fig. 1:**
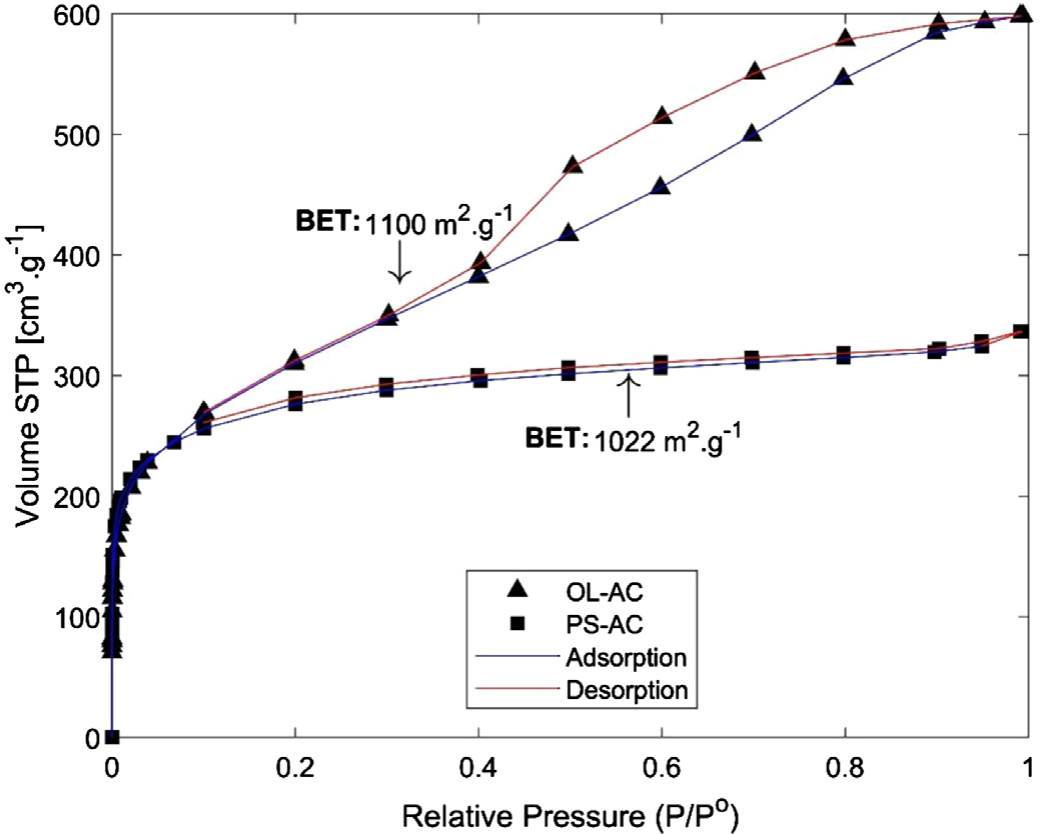
Nitrogen (N_2_) physisorption isotherms at 77 K, and BET surface area.

**Fig. 2:**
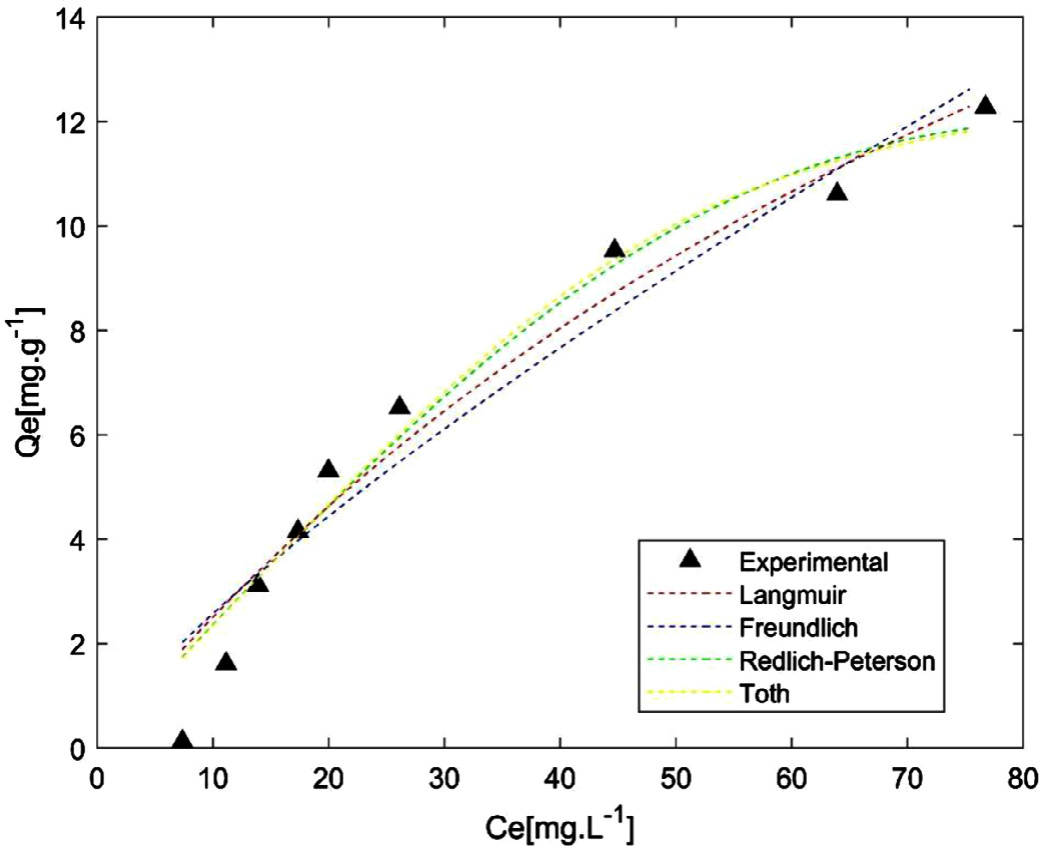
Phenol adsorption isotherms on OL-AC.

**Fig. 3:**
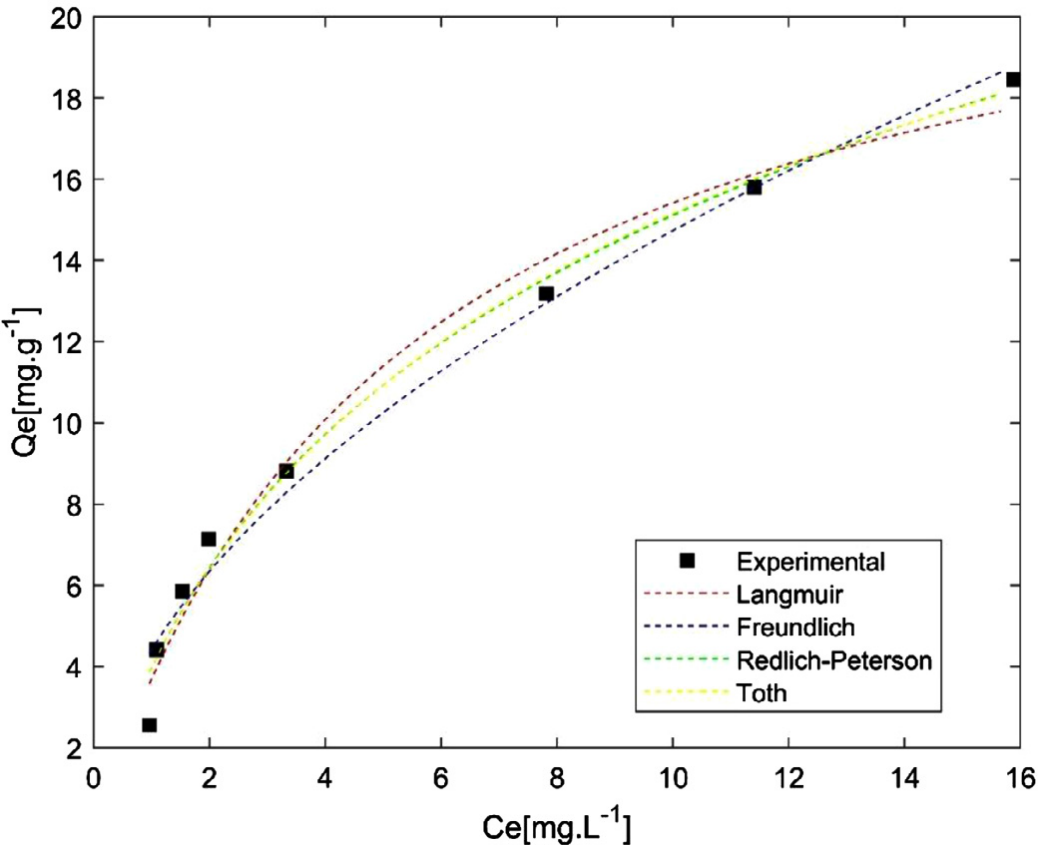
Phenol adsorption isotherms on PS-AC.

**Fig. 4:**
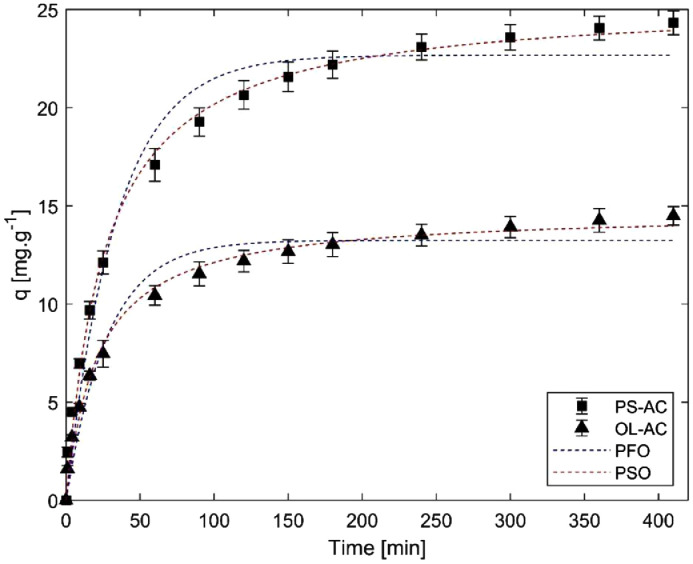
Adsorption kinetics of phenol on AC.

**Fig. 5:**
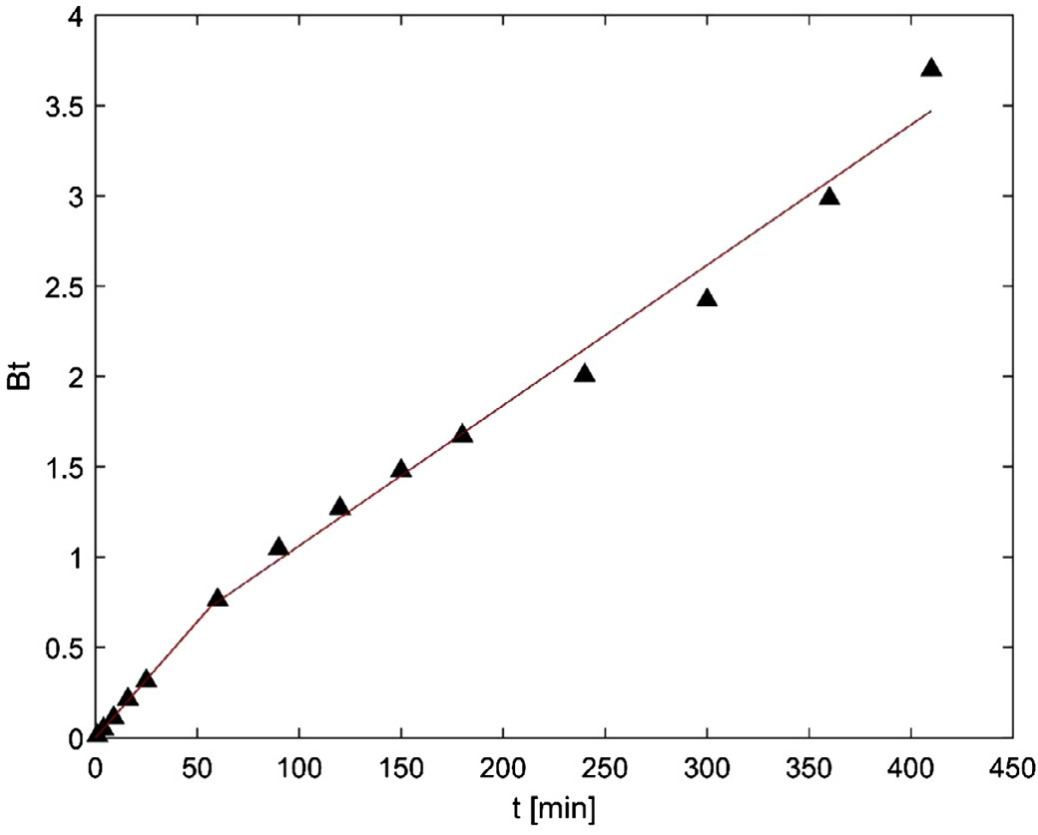
Boyd model for OL-AC.

**Fig. 6:**
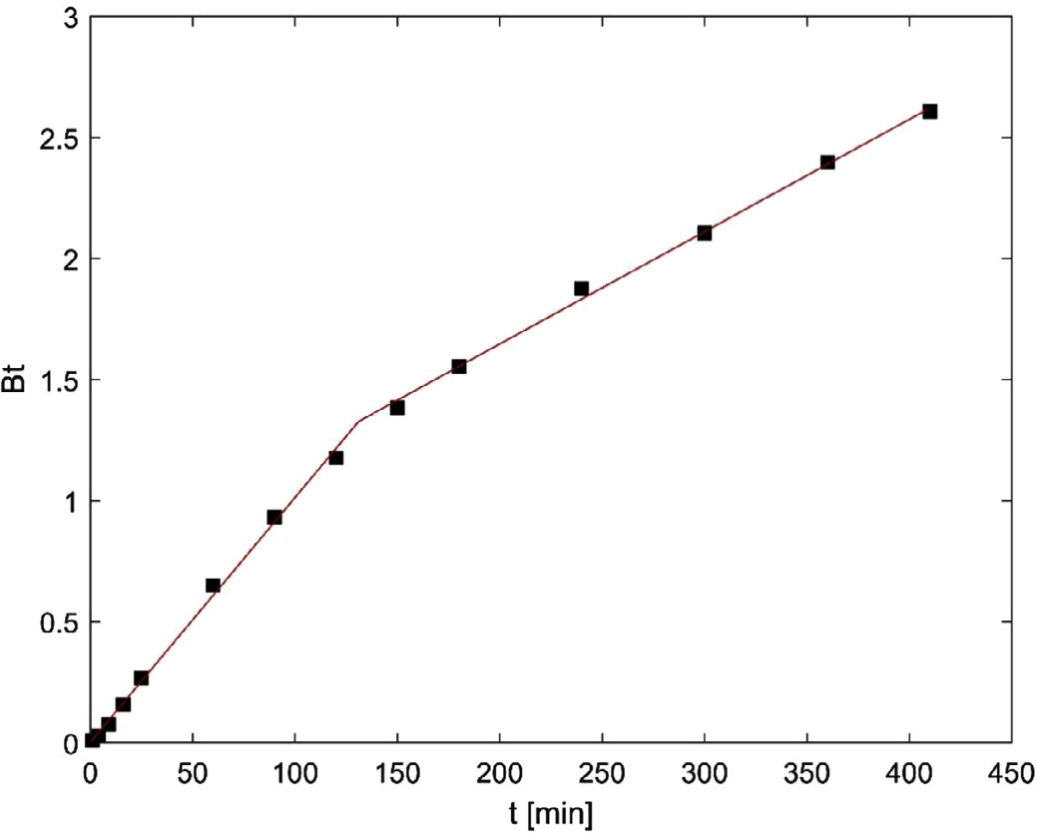
Boyd model for PS-AC.

**Fig. 7:**
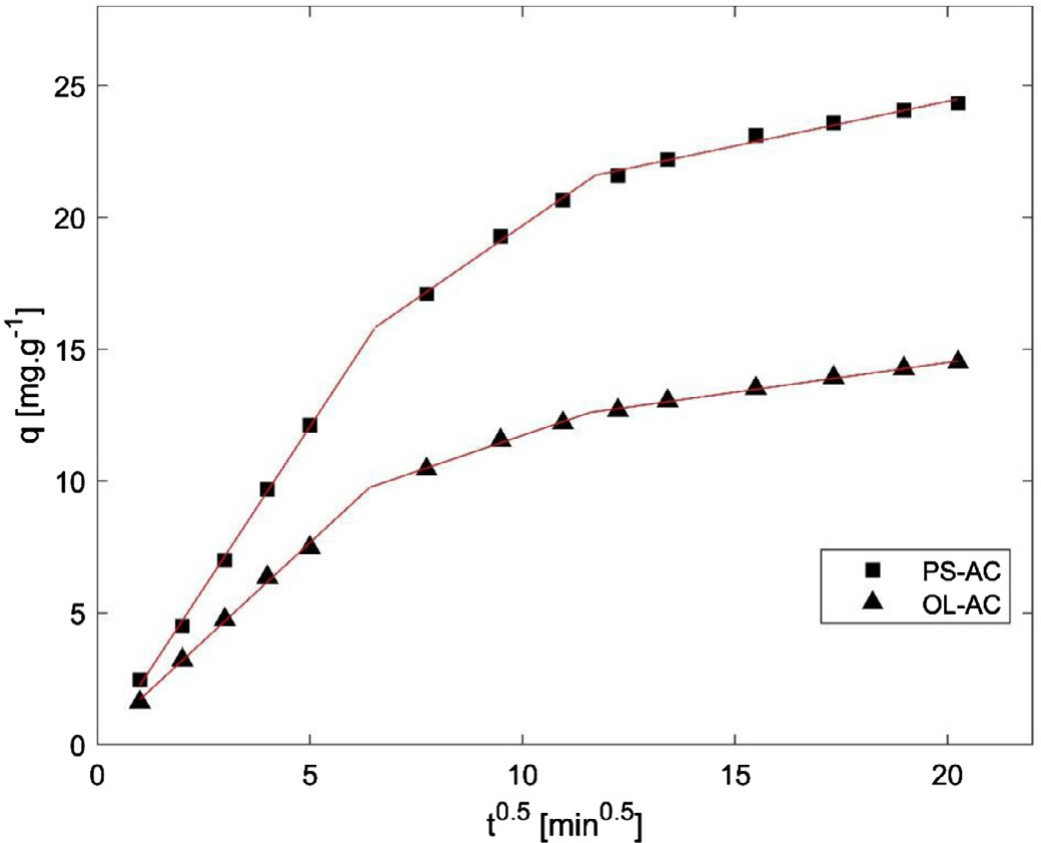
Intraparticle Diffusion model for AC.

These data correspond to adsorption study of phenol over two different activated carbons and the raw data is presented in the *Supplementary File*. BET surface areas were calculated from nitrogen physisorption at 77 K data. Results are presented in Fig. 1. Phenol adsorption isotherms were made to study adsorption-desorption equilibrium, using different isotherm models such as Langmuir, Freundlich, Toth and Redlich-Peterson. These results are presented in Figs. 2 and 3, and the parameters calculated for each model are presented in Table 1. Otherwise, adsorption kinetics was studied to determine information related to adsorption rates. Results can be observed in Fig. 4 and the parameters of PFO and PSO models can be found in Table 2. Boyd and intraparticle diffusion were used to predict the adsorption mechanism and their plots are presented in Fig. 5, Fig. 6, Fig. 7, respectively, while the parameters calculated for both models are presented in Table 3.

**Table 1:** Isotherm models parameters.

Model (Equation)	Parameter	Material	
		OL-AC	PS-AC
*Langmuir* $\left(q_{e}=\frac{q_{m}K_{L}C_{e}}{1+K_{L}\text{Ce}}\right)$	q_m_ [mg.g^−1^]	30.51	23.82
	K_L_ [L.mg^−1^]	0.01	0.18
	SSE [-]	6.41	3.58
Freundlich $\left(q_{e}=K_{F}C_{e}^{\frac{1}{n}}\right)$	K_F_ [L.mg^1–1/n^.g^−1^]	0.42	4.41
	n [-]	1.27	1.91
	SSE [-]	8.76	4.38
*Toth* $\left(q_{e}=\frac{q_{m}K_{T}C_{e}}{{\left(1+{\left(K_{T}C_{e}\right)}^{n}\right)}^{\frac{1}{n}}}\right)$	q_m_ [mg.g^−1^]	12.74	40.54
	K_T_ [L.mg^−1^]	0.02	0.18
	n [-]	3.57	0.56
	SSE [-]	4.88	2.83
Redlich-Peterson $\left(q_{e}=\frac{AC_{e}}{1+BC_{e}^{\beta }}\right)$	A [L.g^−1^]	0.24	5.98
	B [L.mg^−1^]	2.0 × 10^−5^	0.505
	β [-]	2.39	0.77
	SSE [-]	5.12	2.71

**Table 2:** Kinetic models parameters.

Model (Equation)	Parameter	Material	
		OL-AC	PS-AC
*PFO* $\left(q_{t}=q_{e}*\left(1-e^{-k_{1}*t}\right)\right)$	q_e_ [mg.g^−1^]	13.24	22.68
	*k_1_* [min^−1^]	0.04	0.03
	SSE [-]	43.59	88.17
*PSO* $\left(q_{t}=\frac{k_{2}{*q}_{e}^{2}*t}{1+k_{2}*q_{e}*t\;}\right)$	q_e_ [mg.g^−1^]	14.71	25.47
	*k_1_* [g.mg^−1^.min^−1^]	0.003	0.001
	SSE [-]	15.70	24.86

**Table 3:** Boyd an intraparticle diffusion parameters. (a: intercept, b: slope).

Model (Equation)	Parameter	Material	
		OL-AC	PS-AC
*Boyd* $F=\frac{q}{q_{e}}$ $B_{t}=-0.4977-\ln\left(1-F\right)\;\left(F>0.85\right)$ $B_{t}={\left(\sqrt{\pi }-\sqrt{\pi -(\frac{\pi^{2}F}{3})}\right)}^{2}\;\left(F<0.85\right)$	a_1_	−0.003	0.002
	b_1_	0.013	0.010
	a_2_	0.28	0.72
	b_2_	0.008	0.005
Intraparticle diffusion $q_{t}=k_{i}*t^{0,5}+C$	a_1_ (C)	0.21	−0.20
	b_1_ (k_i_)	1.49	2.45
	a_2_ (C)	6.23	8.53
	b_2_ (k_i_)	0.55	1.11
	a_3_ (C)	9.98	17.61
	b_3_ (k_i_)	0.22	0.34

## 2 Experimental design, materials, and methods

### 2.1 Activated carbons preparation and characterization

Activated carbons were prepared from two different lignocellulosic sources. Onion leaves residues (OL) were obtained from local restaurants in Cundinamarca, Colombia, and they were washed and dried at 353 K in an oven. Then they were impregnated with phosphoric acid (H_3_PO_4_) with an impregnation ratio of 6 mmol per gram of OL. Later a thermal treatment (pyrolysis) was carried out using a horizontal tube furnace Thermolyne 79,300 with the following conditions: Maximum temperature of 723 K for two hours and nitrogen atmosphere with a flow of 80 mL.min^−1^. Finally, the onion leaves’ activated carbon (OL-AC) was washed with hot distilled water until neutral pH was reached. In the case of the other precursor, palm kernel shell (PS) was obtained from Cesar, Colombia. Experimental conditions and procedure were the same used for OL-AC but changing the activated agent from H_3_PO_4_ to zinc chloride (ZnCl_2_). This carbon is labelled as PS-AC.

In order to make the surface characterization by nitrogen physisorption isotherms at 77 K, samples were previously degasified at 473 K and vacuum, using an Autosorb IQ2 Quantachrome Instruments. Brunauer-Emmett-Teller (BET) equation was applied to experimental isotherm data to calculate the surface specific area. Results are presented in Fig. 1.

### 2.2 Adsorption isotherms

A stock solution of phenol (Sigma Aldrich, 99% purity) was prepared dissolving 1.0 g in 1.0 L of distilled water, to obtain a solution of 1000 ppm. Then dilutions were made to get solutions with concentrations between 5 and 200 ppm. Adsorption was carried out at 308 K using an AC dosage of 2.5 g.L^−1^ and constant stirring. After two days, adsorption-desorption equilibrium was reached, and the solutions were filtered with PTFE (0.22 μm) filters. Phenol concentration in filtrate was determined by UV–Vis spectrophotometry (Agilent Technologies Cary100) at a maximum absorption wavelength of 270 nm. Adsorption capacities were calculated by a mass balance described by the following equation

$$q_{e}=\frac{\left(C_{0}-C_{e}\right)*V}{m}$$

Where q_e_ is the adsorption capacity at equilibrium [mg.g^−1^], C_0_ is the initial phenol concentration in solution [ppm], C_e_ is the phenol concentration at equilibrium in solution [ppm], V is solution volume [L] and m is the mass of each AC [g]. Experimental data were fitted to four isotherm models: Langmuir, Freundlich, Toth and Redlich-Peterson [1] using nonlinear regression, with an iterative algorithm to minimize the error sums of squares (SSE). The statistical software used was Minitab17. Results are presented in Figs. 2 and 3 and Table 1.

### 2.3 Adsorption kinetics and mechanism

A phenol solution of 100 ppm was used to study adsorption kinetics. Adsorption was carried out at 308 K, using a dosage of 2.5 g.L^−1^ and constant stirring. Samples were taken at minutes 1, 4, 9, 16, 25 and 60 and every half an hour until seven hours passed. Adsorption capacity was calculated for each time using the same equation of the mass balance. Kinetics models PFO and PSO were adjusted to experimental data [2], by nonlinear regression using the statistical software Minitab17, which use an iterative algorithm to minimize the error sums of squares (SSE). Results can be observed in Fig. 4 and Table 2**.**

On the other hand, the adsorption mechanism was studied applying Boyd and Intraparticle Diffusion models. To avoid subjectivity related to the determination of the linear region of both plots, piecewise linear regression was applied using Microsoft Excel and the methodology described by Malash and El-Khaiary [3]. In general, a piecewise linear regression can be expressed with the following equations.

$$Y=a_{1}+b_{1}X,\;X\leq J_{1}$$

$$Y=a_{2}+b_{2}X,\;J_{2}\geq X\geq J_{1}$$

$$Y=a_{n}+b_{n}X,\;X\geq J_{n}$$

Where a_n_ and b_n_ are the intercept and slope of each linear segment, and J_n_ are the breakpoints. This equation system can be solved in Excel using the expressions:

Y=A+BX+C(X−D)SIGN(X−D)

Y=A+BX+C(X−D)SIGN(X−D)+E(X−F)SIGN(X−F)

Where *Y* is the dependent variable, *X* is the independent variable and *A, B, C, D, E and F* are parameters estimated by nonlinear regression. The first equation is used for plots with two linear segments, while the second one is used for plots with three linear segments. The regression parameters (*A, B, C, D, E and F*) are used to calculate breakpoints, slopes and intercepts of each linear segment. The results of Boyd and Intraparticle diffusion models are presented in Fig. 5, Fig. 6, Fig. 7.

## Declaration of Competing Interest

The authors declare that they have no known competing financial interests or personal relationships which have, or could be perceived to have, influenced the work reported in this article.

## Appendix Supplementary materials

Image, application 1

Image, application 2
