# Supplementary material for: Dataset on adsorption of phenol onto activated carbons: Equilibrium, kinetics and mechanism of adsorption
Source: Data Brief. 2020 Sep 14;32:106312. doi: 10.1016/j.dib.2020.106312 (PMC7509189; doi:10.1016/j.dib.2020.106312)
Supplement: Supplementary file 2 [file mmc2.docx]

**Dataset on adsorption of phenol onto activated carbons: Equilibrium, kinetics and mechanism of adsorption**

**Diego Felipe Hernández-Barreto^1^, Liliana Giraldo^2^, Juan Carlos Moreno-Piraján^1*^**

^1^Departamento de Química, Facultad de Ciencias, Grupo de sólidos porosos y calorimetría, Universidad de los Andes, Bogotá, Colombia

^2^Departamento de Química, Facultad de Ciencias, Grupo de calorimetría, Universidad Nacional de Colombia, Bogotá, Colombia

**Corresponding author:**

Juan Carlos Moreno-Piraján ([jumoreno@uniandes.edu.co](mailto:jumoreno@uniandes.edu.co))

**Raw Data**

**Fig. 1.** Nitrogen (N_2_) physisorption isotherms at 77K and BET surface area.

| \| **OL-AC** \| \| \| --- \| --- \| \| **Relative Pressure** \| **Volume STP [cm^3^.g^-1^]** \| \| 4.804E-05 \| 70.5923 \| \| 6.416E-05 \| 75.8751 \| \| 8.277E-05 \| 80.1293 \| \| 9.350E-05 \| 82.2206 \| \| 0.0003 \| 104.5852 \| \| 0.0005 \| 115.9406 \| \| 0.0007 \| 121.9119 \| \| 0.0009 \| 127.9222 \| \| 0.0010 \| 129.9957 \| \| 0.0031 \| 155.5627 \| \| 0.0050 \| 167.1794 \| \| 0.0072 \| 176.2297 \| \| 0.0091 \| 182.4864 \| \| 0.0100 \| 185.1524 \| \| 0.0206 \| 207.0806 \| \| 0.0306 \| 219.6481 \| \| 0.0387 \| 227.7386 \| \| 0.1015 \| 268.3950 \| \| 0.1985 \| 309.9560 \| \| 0.2999 \| 346.9033 \| \| 0.3999 \| 382.0182 \| \| 0.4980 \| 417.0782 \| \| 0.5981 \| 455.6275 \| \| 0.6980 \| 499.3582 \| \| 0.7976 \| 546.0036 \| \| 0.8979 \| 583.9209 \| \| 0.9522 \| 593.1008 \| \| 0.9936 \| 598.2297 \| \| 0.9908 \| 597.9943 \| \| 0.9020 \| 591.7138 \| \| 0.8001 \| 578.3477 \| \| 0.7013 \| 550.9850 \| \| 0.6002 \| 513.9966 \| \| 0.5026 \| 473.0585 \| \| 0.4023 \| 393.4683 \| \| 0.3020 \| 350.0612 \| \| 0.1991 \| 312.2964 \| \| 0.0998 \| 269.2132 \| | \| **PS-AC** \| \| \| --- \| --- \| \| **Relative Pressure** \| **Volume STP [cm^3^.g^-1^]** \| \| 1.384E-06 \| 0.0101 \| \| 2.080E-06 \| 0.1235 \| \| 4.743E-05 \| 88.6160 \| \| 6.145E-05 \| 94.8002 \| \| 8.174E-05 \| 100.3476 \| \| 9.281E-05 \| 102.9163 \| \| 0.0003 \| 128.7027 \| \| 0.0005 \| 139.0625 \| \| 0.0007 \| 145.4999 \| \| 0.0009 \| 150.094 \| \| 0.0010 \| 151.4069 \| \| 0.0033 \| 175.2409 \| \| 0.0051 \| 184.5066 \| \| 0.0071 \| 191.3991 \| \| 0.0090 \| 196.5736 \| \| 0.0101 \| 198.9865 \| \| 0.0202 \| 214.2005 \| \| 0.0300 \| 223.691 \| \| 0.0385 \| 229.9121 \| \| 0.0677 \| 244.9929 \| \| 0.1006 \| 256.1721 \| \| 0.2000 \| 276.3603 \| \| 0.2993 \| 287.8416 \| \| 0.4019 \| 295.8231 \| \| 0.4981 \| 301.4492 \| \| 0.5984 \| 306.4189 \| \| 0.6990 \| 310.8455 \| \| 0.7982 \| 315.0087 \| \| 0.8974 \| 319.6519 \| \| 0.9490 \| 324.4016 \| \| 0.9929 \| 336.6294 \| \| 0.9911 \| 336.6317 \| \| 0.9484 \| 328.2276 \| \| 0.9024 \| 322.5373 \| \| 0.7980 \| 318.6654 \| \| 0.6981 \| 314.9614 \| \| 0.5987 \| 311.0409 \| \| 0.4981 \| 306.6658 \| \| 0.3991 \| 300.5178 \| \| 0.2991 \| 292.8402 \| \| 0.2004 \| 281.623 \| \| 0.0995 \| 260.9843 \| |
| --- | --- | --- | --- | --- | --- | --- | --- | --- | --- | --- | --- | --- | --- | --- | --- | --- | --- | --- | --- | --- | --- | --- | --- | --- | --- | --- | --- | --- | --- | --- | --- | --- | --- | --- | --- | --- | --- | --- | --- | --- | --- | --- | --- | --- | --- | --- | --- | --- | --- | --- | --- | --- | --- | --- | --- | --- | --- | --- | --- | --- | --- | --- | --- | --- | --- | --- | --- | --- | --- | --- | --- | --- | --- | --- | --- | --- | --- | --- | --- | --- | --- | --- | --- | --- | --- | --- | --- | --- | --- | --- | --- | --- | --- | --- | --- | --- | --- | --- | --- | --- | --- | --- | --- | --- | --- | --- | --- | --- | --- | --- | --- | --- | --- | --- | --- | --- | --- | --- | --- | --- | --- | --- | --- | --- | --- | --- | --- | --- | --- | --- | --- | --- | --- | --- | --- | --- | --- | --- | --- | --- | --- | --- | --- | --- | --- | --- | --- | --- | --- | --- | --- | --- | --- | --- | --- | --- | --- | --- | --- | --- | --- | --- | --- | --- | --- | --- | --- | --- | --- |

**Fig. 2 and Fig. 3.** Phenol adsorption isotherms

| \| **OL-AC** \| \| \| --- \| --- \| \| **Q_e_ [mg.g^-1^]** \| **C_e_ [mg.L^-1^]** \| \| 0.1284 \| 7.3669 \| \| 1.6174 \| 11.1461 \| \| 3.1193 \| 13.9005 \| \| 4.1541 \| 17.3595 \| \| 5.3102 \| 19.9858 \| \| 6.5204 \| 26.1351 \| \| 9.5289 \| 44.7111 \| \| 10.6072 \| 63.9277 \| \| 12.2770 \| 76.7388 \| | \| **PS-AC** \| \| \| --- \| --- \| \| **Q_e_ [mg.g^-1^]** \| **C_e_ [mg.L^-1^]** \| \| 2.5546 \| 0.9613 \| \| 4.4150 \| 1.0895 \| \| 5.8558 \| 1.5378 \| \| 7.1387 \| 1.9862 \| \| 8.8092 \| 3.3314 \| \| 13.1921 \| 7.8153 \| \| 15.7966 \| 11.4024 \| \| 18.4483 \| 15.8862 \| |
| --- | --- | --- | --- | --- | --- | --- | --- | --- | --- | --- | --- | --- | --- | --- | --- | --- | --- | --- | --- | --- | --- | --- | --- | --- | --- | --- | --- | --- | --- | --- | --- | --- | --- | --- | --- | --- | --- | --- | --- | --- | --- | --- | --- |

**Fig. 4.** Adsorption kinetics

| **Time [min]** | **OL-AC**  **q [mg.g^-1^]** | **PS-AC**  **q [mg.g^-1^]** |
| --- | --- | --- |
| 1 | 1.6057 | 2.4597 |
| 4 | 3.2113 | 4.5010 |
| 9 | 4.7486 | 6.9778 |
| 16 | 6.3543 | 9.6852 |
| 25 | 7.4731 | 12.1193 |
| 60 | 10.4367 | 17.0900 |
| 90 | 11.5385 | 19.2764 |
| 120 | 12.1876 | 20.6514 |
| 150 | 12.6744 | 21.5738 |
| 180 | 13.0331 | 22.1973 |
| 240 | 13.5114 | 23.0941 |
| 300 | 13.9214 | 23.5809 |
| 360 | 14.2630 | 24.0592 |
| 410 | 14.4936 | 24.3239 |

**Fig. 5. And Fig. 6.** Boyd model

| **Time [min]** | **OL-AC**  **Bt** | **PS-AC**  **Bt** |
| --- | --- | --- |
| 1 | 0.0109 | 0.0085 |
| 4 | 0.0465 | 0.0297 |
| 9 | 0.1091 | 0.0760 |
| 16 | 0.2121 | 0.1580 |
| 25 | 0.3132 | 0.2674 |
| 60 | 0.7629 | 0.6498 |
| 90 | 1.0462 | 0.9320 |
| 120 | 1.2698 | 1.1755 |
| 150 | 1.4780 | 1.3854 |
| 180 | 1.6712 | 1.5552 |
| 240 | 2.0058 | 1.8760 |
| 300 | 2.4223 | 2.1057 |
| 360 | 2.9852 | 2.3983 |
| 410 | 3.6989 | 2.6069 |
